# Risk Factors Associated with Increased Mortality among HIV Infected Children Initiating Antiretroviral Therapy (ART) in South Africa

**DOI:** 10.1371/journal.pone.0022706

**Published:** 2011-07-29

**Authors:** Brian C. Zanoni, Thuli Phungula, Holly M. Zanoni, Holly France, Margaret E. Feeney

**Affiliations:** 1 The Ragon Institute of Massachusetts General Hospital, Massachusetts Institute of Technology, and Harvard, Charlestown, Massachusetts, United States of America; 2 Harvard Medical School, Boston, Massachusetts, United States of America; 3 Sinikithemba Clinic and Philani Program, McCord Hospital, Durban, South Africa; 4 Division of Experimental Medicine, University of California San Francisco, San Francisco, California, United States of America; University of Cape Town, South Africa

## Abstract

**Objective:**

To identify demographic and clinical risk factors associated with mortality after initiation of antiretroviral therapy (ART) in a cohort of human immunodeficiency (HIV) infected children in KwaZulu-Natal, South Africa.

**Methods:**

We performed a retrospective cohort study of 537 children initiating antiretroviral therapy at McCord Hospital in KwaZulu-Natal, South Africa. Data were extracted from electronic medical records and risk factors associated with mortality were assessed using Cox regression analysis.

**Results:**

Overall there were 47 deaths from the cohort of 537 children initiating ART with over 991 child-years of follow-up (median 22 months on ART), yielding a mortality rate of 4.7 deaths per 100 child years on ART. Univariate analysis indicated that mortality was significantly associated with lower weight-for-age Z-score (p<0.0001), chronic diarrhea (p = 0.0002), lower hemoglobin (p = 0.002), age <3 years (p = 0.003), and CD4% <10% (p = 0.005). The final multivariable Cox proportional hazards mortality model found age less than 3 years (p = 0.004), CD4 <10% (p = 0.01), chronic diarrhea (p = 0.03), weight-for-age Z-score (<0.0001) and female gender as a covariate varying with time (p = 0.03) all significantly associated with mortality.

**Conclusion:**

In addition to recognized risk factors such as young age and advanced immunosuppression, we found female gender to be significantly associated with mortality in this pediatric ART cohort. Future studies are needed to determine whether intrinsic biologic differences or socio-cultural factors place female children with HIV at increased risk of death following initiation of ART.

## Introduction

Pediatric HIV remains prevalent in sub-Saharan Africa. As of 2009, South Africa alone had more than 280,000 HIV-infected children [1]. Untreated pediatric HIV infection carries a high mortality rate [2], [3]. However, access to ART in sub-Saharan Africa has increased dramatically since 2005 [1], [4], [5], [6]. Until recently, few data were available regarding clinical risk factors associated with mortality in children after they initiate ART [7], [8], [9], [10], [11]. Recent studies from Southern Africa have consistently found low absolute CD4 and CD4% at the time of ART initiation to be associated with early mortality on ART [5], [7], [8], [11]. Additionally, several studies confirmed that stunting, wasting, anemia, and WHO stage 4 conditions at the time of ART initiation significantly contribute to early pediatric mortality on ART [5], [8], [10], [11].

Although, gender has not previously been associated with mortality in these African cohorts, several studies have documented gender discrepancies in response to HIV infection [12], [13], [14], [15]. Two small studies documented lower HIV RNA in girls compared to boys after initiation of ART [13], [14]. However, an additional study found no difference in viral loads between the genders [16].

Expanded knowledge of baseline factors associated with early mortality could improve local guidelines regarding the timing and choice of ART regimen and help target intensity of medical surveillance after ART initiation towards those children at highest risk of early death.

## Materials and Methods

### Ethics Statement

The protocol was approved by McCord Hospital's Research Ethics Committee and the Partners Human Research Committee. All patients accessing care at McCord Hospital sign a written consent to have their medical information stored on an electronic medical record database that is used for clinical and research purposes.

### Study design

We performed a retrospective cohort study using electronic medical records from HIV infected pediatric patients (≤18 years old) who initiated antiretroviral therapy at McCord Hospital's Sinikithemba Clinic in KwaZulu-Natal, South Africa from August 2003 to December 2008. We analyzed clinical and demographic characteristics at the time of initiation of ART therapy as potential predictors of mortality.

### Study population and standard of care

McCord Hospital is a semi-private, urban hospital providing care for a mostly Zulu speaking population in Durban, South Africa. Between August 2003 and December 2008, 537 children initiated ART at McCord Hospital's Sinikithemba Clinic. Patients were followed from the time they initiated ART until they died, transferred care to another facility, were lost to follow-up, or until the study end date of May 31, 2009. In this clinical setting, children were diagnosed with tuberculosis based on a combination of available clinical, radiographic, microscopic, and contact information. During the study period, children were initiated on ART when their HIV disease reached World Health Organization (WHO) stage 3 or 4 and/or their CD4 percentage was less than 20% in children younger than 18 months, or less than 15% in children older than 18 months, in accordance with South African National Treatment Guidelines [17]. Based on national guidelines in South Africa, children less than 3 years of age received a Protease Inhibitor (PI)-based first line treatment regimen comprised of lopinavir/ritonavir, stavudine, and lamivudine [17]. Children older than 3 years initiated a Non-Nucleotide Reverse Transcriptase Inhibitor (NNRTI) based treatment regimen comprised of efaverinez, stavudine, and lamivudine [17]. According to local guidelines routine laboratory monitoring includes baseline CD4 and 6 monthly CD4 and viral loads [17]. Throughout the duration of the study period, children taking an efaverinez based regimen who had HIV-TB co-infection did not have any alterations to their treatment while children receiving protease inhibitor based treatment were either changed from lopinavir/ritonavir to ritonavir or received double dosed lopinavir/ritonavir.

### Data Collection

We evaluated medical records from all patients who were ≤18 years old when they initiated ART at McCord Hospital's Sinikithemba Clinic from August 2003 to December 2008. TrackCare Software was used to maintain electronic medical records, which were cross referenced with paper charts. Data were entered into a Microsoft Access database, and we then reviewed 10% of all medical records for accuracy. Collected data included age at ART initiation, gender, ART regimen, presence of tuberculosis (TB) and non-TB opportunistic infections, chronic diarrhea (longer than 14 days), baseline laboratory results including absolute and CD4 percentage, and hemoglobin, and baseline weight-for-age Z-scores (calculated for all children less than 10 years of age using the WHO macro for STATA; https://www.who.int/childgrowth/software). Children older than 10 years were assigned a dummy variable for missing weight-for-age z-score so that they could be included in the multivariable model. The presence of chronic diarrhea and opportunistic infections were recorded based on their presence in the electronic medical record as well as review of the hard copy paper records. If these conditions were not documented in the electronic medical record or paper charts they were reported as absent.

#### Lost to Follow up, Transfers and Deaths

Children were considered lost to follow up if they did not collect treatment for 3 consecutive months. Attempts were made to contact all children who were lost to follow up to determine their status. All lost to follow ups and transfers were censored on the date of their last visit. Otherwise follow-up time was censored at the end of the study period. Death events were defined as all-cause deaths occurring after ART initiation but before the May 31, 2009 end of the study period. Deaths were confirmed by hospital records or family interview. Cause of death was determined by the primary care physician or by verbal autopsy from family members.

#### Missing Data

In multivariate analysis, only subjects with complete data were analyzed. In univariate analysis subjects were included if they had complete data for the covariate of interest. No data was imputed for this analysis. Since weight-for-age Z-score was systematically missing for children older than 10 years, two variables were included simultaneously in the model for Z-score as a continuous variable and the other as a categorical indicating the absence of Z-score. Including both variable simultaneously allows children >10 years to be included in the multivariable model without imputing values.

### Statistical Analysis

Statistical analyses were conducted using SAS statistical software (Release 9.2, Carey, NC). Time to mortality was assessed for subjects from the date of ART initiation to the date of death occurring before May 31, 2009 (end of the study period).

We used Cox proportional hazards regression models to assess associations between demographic and clinical characteristics and mortality. We first determined univariate associations between nine demographic and clinical covariates that, based upon clinical observations and prior studies, were suspected to be potentially important correlates of post-ART mortality. Age was stratified into 2 categories (<3 years and ≥3 years old) to account for the age related difference in ART treatment regimens. We did not assess the association between type of ART regimen (Protease Inhibitor verses Non-Nucleotide Reverse Transcriptor Inhibitor) and mortality in these analyses because the ART regimen was selected based on age, and thus these variables were highly correlated (Pearson correlation coefficient = 0.83). Gender was added in *a priori* to assess age and sex matched controls. Female gender was found to violate the proportional hazard assumption of the Cox model; therefore, was evaluated as a time varying hazard in the final model. We constructed Kaplan-Meier survival curves stratified by age and gender and assessed the difference between the curves using the log rank test. Since CD4 <15% is required for initiation of ART in most children in South Africa [17], the data was highly skewed; therefore, we stratified the CD4 percent variable at <10% and ≥10%. Using the remaining eight variables assessed in univariate analysis, we constructed a multivariable mortality model and used the Score Test criterion in combination with Akaike Information Criterion (AIC) to determine the most parsimonious model [18].

We evaluated the proportional hazards assumptions for the variables in the final multivariable mortality model using the Schoenfeld residuals method. This tests whether the residuals for each variable are uncorrelated (P>0.05) with the ordering of times to death events. We found that the female gender was significantly correlated with death event times (P = 0.02). The other variables were not significantly correlated with death event times. We therefore further evaluated the proportional hazards assumption for the female gender variable by assessing it within the full multivariable Cox proportional hazards regression model as a time-varying covariate with time considered as natural log of time. This time varying covariate was significant (P = 0.03) after adjusting for the other covariates within the multivariable model.

## Results

### Patient Characteristics and Mortality

Demographic and clinical characteristics of the 537 analyzed subjects are described in Table 1. Forty-seven subjects died during the study period, with deaths occurring a median of 60 days after starting ART (range 5 days–2.7 years). The overall mortality in this cohort was low, with only 4.7 deaths per 100 child years on ART. Of the 490 surviving subjects, 18 (3.7%) (10 females, 8 males) were lost to medical follow-up and 72 (14.7%) (41 females, 31 males) subjects were no longer followed because they transferred care to another clinic. Median follow-up for the surviving subjects was 22 months, with follow-up ranging from 14 days to 5.3 years.

**Table 1:** Demographic and Clinical Characteristics.

Baseline Characteristics	N Total	Complete Cohort	Males	Females	p-value
**N**	537		261 (48.6%)	276 (51.4%)	
**Age at ART initiation (median; years) [IQR]**	537	6.0 [2.4–9.3]	5.95 [2.23–8.75]	6.25 [3.1–9.7]	0.08
**<3 years**		149 (27.8%)	80 (30.7%)	69 (25%)	0.15
**3–18 years**		388 (72.2%)	181 (69.3%)	207 (75%)	
**CD4 absolute (median; cells/µL)[IQR]**	535	226 [70–447]	242 [80–467]	205 [69–438]	0.50
**CD4 <10%**	524	250 (47.7%)	126 (49.4%)	124 (46.1%)	0.48
**Documented OI at baseline**	537	138 (25.7%)	63 (24.1%)	75 27.2%)	0.43
**Chronic Diarrhea**	537	112 (20.9%)	59 (22.6%)	53 (19.2%)	0.34
**Hemoglobin (median; g/dl)[IQR]**	482	10.0 [8.9–11]	10 [8.8–10.9]	10.1 [8.9–11.1]	0.37
**Weight-for-age z-score** † **(median) [IQR]**	403	−1.4 [−2.6–−0.5]	−1.5 [−2.8–−0.6]	−1.3 [−2.4–−0.5]	0.14
**TB at initiation of ART**	537	228 (42.5%)	107 (41.1%)	121 (43.8%)	0.54
**NNRTI treatment** *		414 (77.1%)	195 (74.7%)	219 (79.4%)	0.22
**Deaths**		47 (8.8%)	19 (7.3%)	28 (10.1%)	0.29

*20 children in the <3 year old group were started on NNRTI while all children over 3 years old were initiated on NNRTI based treatment.

† WHO weight-for-age Z-score is only valid for children <10 years old.

Most of these deaths occurred early; 64% (30) occurred within 3 months, and 83% (39) within 6 months of ART initiation. Only 3 children died after 12 months of ART, accounting for 0.56% of the entire cohort with a total follow up time in the study of over 991 child-years. Of the early deaths that occurred before 6 months on ART,28% (11) died of tuberculosis and 18% (7) died of diarrhea, dehydration or malnutrition. Among the children who died between 6 months and one year after initiation of ART, all 5 had plasma HIV RNA levels of <400 copies/ml measured after 6 months on ART. The cause of death was determined to be culture confirmed tuberculosis in 2 children (40%) (both with multiple episodes of tuberculosis treatment), severe malnutrition with chronic diarrhea in 2 children (40%) and unknown in 1 child (20%). Of the 3 children that died after completing one year of ART, 2 (66%) died with high levels of viremia on second-line ART and 1 child died of disseminated tuberculosis.

### Univariate Assessment of Mortality Risk

**Figure 1:**
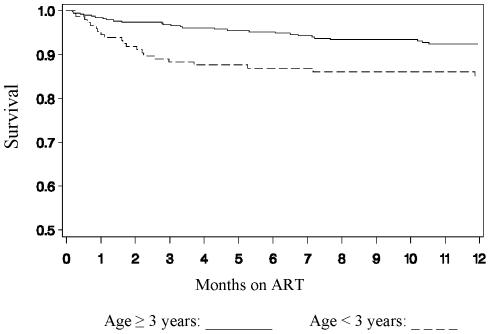
The age-stratified Kaplan-Meier graph of survival after ART initiation.

Univariate associations between demographic and clinical characteristics and mortality are shown in Table 2. Weight-for-age Z-score (p<0.0001), chronic diarrhea (p = 0.0002), lower hemoglobin (p = 0.002), age <3 years (p = 0.003), and CD4% <10% (p = 0.005) were all strongly associated with mortality. The age stratified Kaplan Meier survival curves for time after initiation of ART are shown in Figure 1. Non-TB opportunistic infections (p = 0.04) and absolute CD4 count (p = 0.05) were also significantly associated with death.

**Table 2:** Unadjusted Cox Proportional Hazard Ratios for Mortality after Initiating HIV Treatment.

Demographic and Clinical Variables† n = 537	Hazard Ratio (95% Confidence Interval) 47 death events	P value
Age <3 years	2.42 (1.37–4.30)	0.003
Female gender	1.43 (0.80–2.56)	0.23
Absolute CD4 count (cells/µL)	0.999 (0.998–1.000)	0.05
CD4 <10%	2.49 (1.33–4.66)	0.005
Hemoglobin (gm/dL)	0.76 (0.63–0.90)	0.002
Opportunistic infection not tuberculosis	1.85 (1.03–3.33)	0.04
Tuberculosis at or after initiation of HIV therapy	1.63 (0.92–2.92)	0.10
Chronic diarrhea	3.02 (1.69–5.39)	0.0002
Weight-for-age Z-score	0.53 (0.43, 0.64)	<0.0001

† *Absolute CD4, hemoglobin* and *weight-for-age Z-score* were treated as continuous variables. Reference groups for the binary variables include: *Age <3 years* – reference age 3–18 years; *CD4 <10%* - reference CD4 >10%, *Opportunistic infection* includes children with the presence of candidiasis, Kaposi's sarcoma, Pneumocystis jiroveci pneumonia, cryptococcosis, toxoplasmosis, or cryptosporidiosis, while the reference group did not have any of these opportunistic infections; *Tuberculosis at or after initiation or HIV therapy* – reference was the absence of HIV-tuberculosis co-infection; *chronic diarrhea* – reference children not documented to have chronic diarrhea.

### Multivariable Assessment of Mortality Risk

**Figure 2:**
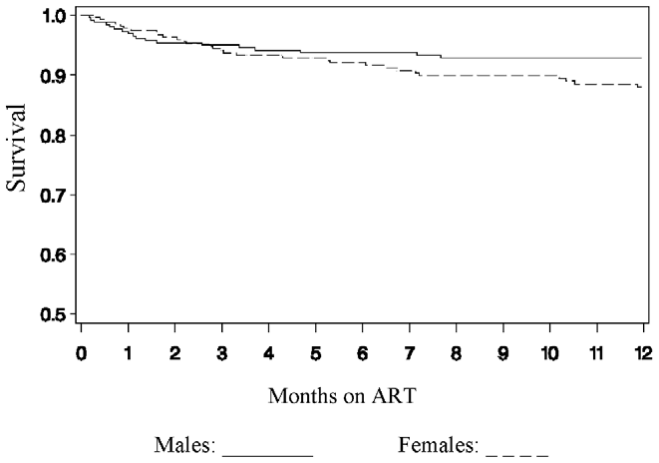
The gender-stratified Kaplan-Meier graph of survival after ART initiation.

A multivariable Cox proportional hazards mortality model was developed using the following covariates: weight-for-age Z-score, chronic diarrhea, hemoglobin, CD4%, tuberculosis coinfection, presence of opportunistic infection, age <3 years, and gender. The final model, selected from the best fit model with the lowest AIC using the Score Test [13], is shown in Table 3. In multivariate analysis, age, CD4 <10%, chronic diarrhea, and weight-for-age Z-score were all shown to be strongly and independently predictive of mortality. Interestingly, this analysis also revealed a difference in the survival rate between males and females following initiation of ART. Although female gender was not significantly associated with mortality in univariate analysis, it was significantly associated (p = 0.03) with mortality when controlling for age, weight-for-age Z-score, chronic diarrhea, and CD4%. The unadjusted Kaplan-Meier graph is illustrated in Figure 2. Female gender was best described as varying linearly with the natural log (ln) of time within the multivariable model.

**Table 3:** Adjusted Cox Proportional Hazard Ratios for Mortality after Initiating HIV Treatment.

Predictors † n = 524*	Hazard Ratio (95% Confidence Interval) 46 death events	P value
Age <3 years	2.66 (1.37–5.17)	0.004
CD4 <10%	2.39 (1.21–4.71)	0.01
Chronic diarrhea	1.91 (1.05–3.49)	0.03
Weight-to-age Z-score	0.59 (0.47–0.73)	<0.0001
Female*ln time‡	1.79 (1.05–3.49)	0.03

† *Weight-for-age Z-score* and Female*ln time were treated as continuous variables. Reference groups for the binary variables include: *Age <3 years* – reference age 3–18 years; *CD4 <10%* - reference CD4 >10%; *chronic diarrhea* – reference children not documented to have chronic diarrhea.

*13 subjects were missing one or more of the model's predictor variables and were not included in the analysis.

‡ Female*ln time represents the interaction term of female gender and the natural log of time in weeks since ART initiation.

### 6 Month Viral Response

At six months of ART therapy there were 424 subjects with viral load data available. There was no significant difference (p = 0.56) in viral suppression rates to <400 copies/ml between males 170/198 (86%) and females 199/226 (88%).

### Sensitivity Analysis

In order to determine the impact of lost to follow-up, a sensitivity analysis was performed including the composite endpoint of lost to follow-up and death. This did not significantly change any results in the univarite analysis. In the multivariate analysis only the presence of chronic diarrhea was no longer significant with a p value of 0.12. Age less than three years (p = 0.006), CD4 <10% (p = 0.016), weight for age Z-score (p<0.001), and female gender (p = 0.007) all remained significant.

## Discussion

The increased availability of ART in sub-Saharan Africa has dramatically improved outcomes for pediatric patients infected with HIV. However, treatment guidelines and algorithms continue to evolve, and there is a pressing need for clinical outcomes data to further refine HIV management strategies for children in resource-limited settings. There is a particularly pressing need for data regarding clinical factors that place these children at increased risk of early mortality despite initiation of ART. Our findings agree with published data from other African cohorts that age <3 years, severely reduced CD4 percent (<10%), chronic diarrhea, and reduced weight-for-age Z-scores are strongly associated with mortality among HIV positive children initiating ART [5], [7], [8], [9], [10], [11], [19], [20].

The mortality risk factors identified in this study highlight several areas for potential improvement in pediatric treatment and monitoring algorithms. The increased mortality in younger children and those with low CD4 plus the growing body of evidence of improved outcomes in infant ART initiation argues for the early initiation of ART in HIV positive children [21]. In response, WHO treatment guidelines have recently been altered to recommend expansion of ART to all infants younger than 24 months and all young children with CD4 percentages lower than 25% [22]. Importantly, the vast majority of deaths in our cohort occurred prior to the first set of monitoring labs, which are drawn after six months of therapy per South African guidelines. It would seem prudent to target children with multiple risk factors to receive earlier clinical and laboratory follow-up to ensure an adequate response to therapy. The increased mortality in children with chronic diarrhea and low weight-for-age Z-score at the time of initiation of ART indicates the importance of monitoring nutritional status at the time of ART initiation and argues for earlier ART initiation in these children. Future studies on early aggressive nutritional monitoring and interventions could lead to improved outcomes in children with malnutrition or chronic diarrhea. An unexpected finding from our study was the increased risk of mortality observed among females after controlling for other risk factors. The mortality risk associated with female gender increased progressively with time after initiation of ART therapy. It is unclear whether this disparity is attributable to intrinsic biological differences between the sexes [13], [14], [23], [24], [25] or social differences such as delayed presentation for care, differences in parental supervision of boys and girls, or devaluation of the female gender that could place females at a higher risk of mortality after ART initiation in South Africa. Among adults, females tend to have lower viral loads early in HIV-1 infection but progress faster to AIDS for a given viral load than males [15], [25], [26]. Therefore, it has been suggested that initiating HAART based on CD4 and viral load criteria without accounting for gender differences may disadvantage women [25]. Although pediatric data are limited, two prior studies have suggested gender differences in HIV viral loads among children [13], [14], which could provide a biologically plausible basis for our observation of a gender difference in survival on HAART. This finding has not been seen in other HIV mortality analyses of African children [5], [7], [8], [9], most of which utilized smaller cohorts or pooled data from multiple centers. However, a similar increase in mortality was seen among female children in the Women and Infants Transmission Study (WITS), a longitudinal study of HIV-infected children in North America [14]. In addition, the European Collaborative group found more rapid disease progression in HIV infected females less than 4 years old [13]. The published data regarding the impact of gender on survival in HIV-infected adults are significantly confounded by differences in healthcare utilization, as several North American studies have reported lower healthcare utilization and viral suppression rates among women [27], [28], [29], while several African studies have reported better health utilization and outcomes among HIV positive women on ART [30], [31], [32], [33], [34]. However, data from adult studies may not be generalizable to young girls, who are dependent on adult caregivers for accessing clinic services. Therefore, local societal devaluation of female children may place them at unique risk for poor outcomes on ART.

Although tuberculosis co-infection was not significantly associated with mortality in this analysis, this cohort did exhibit a high incidence of tuberculosis infections and TB was a major contributor to deaths. The high incidence of TB may represent over diagnosis and overlap with lymphocytic interstitial pneumonitis [35]. Given the clinical setting many of these were not bacteriologically confirmed cases tuberculosis; however, represented clinical diagnosis based on symptoms, radiography, and contact information. This highlights the pressing need for improved diagnostics for pediatric tuberculosis infections.

One limitation of our study is that it assesses a single cohort of HIV positive children from KwaZulu-Natal, South Africa, and a relatively small number of subjects (8.8%) died during the study period. It is possible that larger studies with increased statistical power may identify other risk factors that significantly predict mortality after initiation of ART in pediatric HIV patients. Given the small number of children lost to follow-up (LTFU), sensitivity analysis using the composite endpoint of LTFU and mortality did not significantly alter the major findings of this study. In addition, this study was located in a semi-private institution which had nominal user fees of R50 (approximately $7 USD) per month which could result in a selection bias toward a wealthier population. In contrast to many sub-Saharan pediatric populations, our cohort generally had access to daily meals and water, as well as medical care at a semi-private clinic, and thus our results may not be generalizable to more impoverished settings.

Further studies are needed to define gender differences in the biological manifestations of HIV infection in boys and girls, and to determine the relationship between gender and survival among children receiving treatment for HIV infection. Our expanding knowledge of the risk factors associated with mortality in HIV infected children on ART can help to further refine HIV treatment guidelines and monitoring practices.
